# Supplementary material for: Chromothripsis during telomere crisis is independent of NHEJ, and consistent with a replicative origin
Source: Genome Res. 2019 May;29(5):737–49. doi: 10.1101/gr.240705.118 (PMC6499312; doi:10.1101/gr.240705.118)
Supplement: Supplemental Material [file supp_gr.240705.118_Supplemental_file_1.zip › contigs/annotated_contigs/DB53/contig.2.DB53_length_514_mean_cov_3.60700389105.docx]

**DB53_length_514_mean_cov_3.60700389105**

```
ATTACCAAATCTGCCTGTTTAAGGAAAGGCACAAATTCAGATTGTTAATGTGAAATCTATTGACTTGTAAGTGTTGGCACCTATTTTTA
 >chr8:128734814-128734994 + E=1e-96
AATGTTATAAATGCTGAGAGGGTCAAAACCTGTCATCCAAGCCAACCTACCAGTAAGCAAGACTTGGTCCTCAAGCAAGTTTGCTGCCT

C|T|AACCCAAGAAGATATTAAAATAGAAATTAATCTTTGGAGCTTTTGCTTCATTGACCTCTAACCCTTACTGTAATCAGGAGGAAAT
  >chr8:131154299-131154634 - E=4e-190
GTTCTTGTGTGCTGGGCATCCAGCTGTTATTCTCCGAGCTATATCAGATTACTTTCTTTCCAGGCTGGACTGCAGAGTGACCGCAGAAG

AGAAGGAGTGGGGGCTCTGACCCATCCAAACCACTTTCTTACCGGGAGAGCTTAGGAATGTTACTTAACCTTTCTGTGCCTTTATTTCT

TCACTTAGTAAAAGAAACTGAAGACTTCCCTCACAGGTAGTACATGAGATAGTATATGACATAAGATGCCT
```
